# Supplementary material for: Facilitators and barriers of relatives’ involvement in nursing care decisions and self-care of patients with acquired brain injury or malignant brain tumour: A scoping review protocol
Source: PLoS One. 2022 Aug 18;17(8):e0273151. doi: 10.1371/journal.pone.0273151 (PMC9387856; doi:10.1371/journal.pone.0273151)
Supplement: Attachment — Submitted filename: Review.docx [file pone.0273151.s001.docx]

**Review report on protocol title: Facilitators and barriers towards nurses’ involvement of relatives: a scoping review 2 protocol, manuscript PONE-D-21-20624.**

1. **Topic:** The topic is clearly writing and significant to undertake the study. It is also scientifically sound as it contains the population to be study and its comparison which is the patients.
2. **Abstract:** The abstract is also well writing and included the registration number of the protocol.
3. **Introduction:** the background is very detailed, informative, and well writing. The rational of the study is also included “to investigate possible facilitators and barriers towards nurses’ involvement of relatives to individuals with acquired brain injury or malignant brain cancer in all settings”. Previous literature used are also referenced.
4. **Methods and materials:** the objective of the study is well writing, specific and in simple terms. The study design aim to be used is systematic review which is recommended to provide highest source of evidence.

- They have stated the inclusion criteria which is “studies looking at nurses caring for patients in acquired brain damage in all settings”. However, the exclusion criteria are not stated in the protocol which might be important to guide those doing the extraction process.
- They have also stated in the methods how search strategy is to be conducted in steps and the sources of information. Data extraction was indicated will be done by two independent extractors and third arbitrator will be involve in case of disagreement.

5. **Data presentation:** it is stated that the data presentation will be done in a tabular form followed by narrative summary.
6. **Overall: I consider the study significant and recommend its commencement because the study is relevant to healthcare professionals to break the barriers of not involving relatives in patients’ management.**
