# Supplementary material for: Facilitators and barriers of relatives’ involvement in nursing care decisions and self-care of patients with acquired brain injury or malignant brain tumour: A scoping review protocol
Source: PLoS One. 2022 Aug 18;17(8):e0273151. doi: 10.1371/journal.pone.0273151 (PMC9387856; doi:10.1371/journal.pone.0273151)
Supplement: Attachment — Submitted filename: 110222 Response_to_Reviewers.docx [file pone.0273151.s002.docx]

**Title of the Manuscript**: Facilitators and barriers towards nurses’ involvement of relatives: a scoping review protocol

**Manuscript Number**: PONE-D-21-20624

***Reviewer 1***

**Comment 1**: *IMPORTANT* Scoping reviews are underdone systematic reviews. This is seen as an excuse to cut corners and provide substandard processes, all in an effort to privilege speed, cost, or some other non-pertinent reason. A review is either performed systematically or it is not. In the present case, the protocol misses out on several important and crucial methodological steps. If followed, the resulting research will be substandard and inutile.

Authors’ Response: Thank you for the comment. However, we argue as Munn and Levac that scoping reviews are useful for exploring emerging evidence when it is still unclear what other, more specific questions can be posed and valuably addressed by a more precise systematic review (1, 2). We agree that a scoping review need to be rigorously conducted, transparent and trustworthy. To ensure this we refer to the JBI’s Guidance for the conduct of a Scoping Review(3). We have rewritten the method section to make this clearer.

**Change to Text**:

We will conduct a scoping review to identify and map the evidence concerning the involvement of relatives in patients with ABI or MBT in accordance with the Joanna Briggs Institute’s (JBI) methodology for scoping reviews. A scoping review is suitable for our topic area as scoping reviews is defined as ‘a form of knowledge synthesis that addresses an exploratory research question aimed at mapping key concepts, types of evidence, and gaps in research related to a defined area or field by systematically searching, selecting and synthesizing knowledge’ .^(4)^ In our scoping review at least two independent reviewers will participate in an iterative process of screening the literature, paper selection and data extraction. Disagreements between the reviewers will be resolved by discussion until consensus is reached or after consultation with the research team if needed. Results will be reported with descriptive statistics and diagrammatic or tabular displayed information, accompanied by narrative summaries as explained in the JBI guidelines (ref).

**Comment 2**: First, the authors provide no process of synthesis. What theories or frameworks guide their assessment of the evidence? Without a process of synthesis, then the resulting information is no better than a tally or a list of facilitators and barriers. This is hardly research, since the authors will simply be regurgitating information found in the feeder studies. A prime example of this is the dummy table provided by the authors as a guide to the presentation of results. This table is trivial as it simply lists each item tallied by the authors. There is no effort to apply a framework to the results. The nursing literature is replete with significant frameworks, theories or models on which to synthesise the results. That the authors have not cited any is quite disturbing.

**Authors’ Response**:

Thank you for your comment. Please see the comment above.

**Comment 3**: Second, the authors do not provide a method of assessing the quality of the studies. The assumption they are using, in effect, is that all the studies were conducted equally well. This is ridiculous.

**Authors’ Response**:

Thank you for your comment. According to Munn et.al (2018) It is not within the remit of a scoping review to assess	quality of the study or to produce a critically appraised answer to a particular question(1). As Munn states ‘An assessment of methodological limitations or risk of bias of the evidence included within a scoping review is generally not performed’(1). The scoping review methodology is chosen because it is suitable when the aim is to identify and map the available evidence concerning on what kind of possible facilitators and barriers towards nurses’ involvement of relatives to individuals with acquired injury or malignant brain tumour.

**Comment 4**:
Third, the authors do not provide a method of understanding the degree of differences between the studies. An example of such differences is context. The involvement of relatives by nurses is highly contextual. For example, Hospital A might have existing programs designed to involve relatives in patient care. Thus, data from Hospital A will list a set of facilitators and barriers that will be quite different from other settings and will be quite inapplicable to others because it depends on the prior existence of such programs. The existence of such environmental factors have to do with the context of the research. This is undefined and ignored.

**Authors’ Response**:

Thank you for your comment. As we are interested in what kind of possible facilitators and barriers towards nurses’ involvement of relatives through the course of disease to individuals with acquired injury or malignant brain tumour we will include studies from all settings. To be able to identify possible environmental factors that may influence facilitators and barriers for involvement we have added context to expected extraction fields on P 5. L 180 and to the data presentation template and will extract data related to context/setting (ICU, in-outpatient clonic, primary health care, nursing home ect.).

***Reviewer 2***

**Comment 1**: The title is incomplete.. involvement of relatives in what?

**Authors’ Response**: Thank you for this comment. We have been more specific about the ‘what’

**Change to Text**: Facilitators and barriers towards nurses’ involvement of relatives in decision making and daily life activities through the course of disease of patients with an acquired brain injury or malignant brain tumour.: a scoping review protocol

**Comment 2**: The abstract is not organized. you start with introduction, then objectives. inclusion criteria should be part of the methods.

**Authors’ Response**: Thank you for this comment. The abstract is now organized

**Change to Text**:

**Comment 3**: Write in full first before abbreviating - see under introduction, line 1, TBI, ABI,

Author’s Response: Thank you for pointing this out. This have been corrected on line 1 and line 35

**Change to Text**:

**Comment 4**: Consider using the term participation rather than involvement. relatives may be involved but not participate in the patient care.

Author’s Response: Thank you for your suggestion. As stated on P. 2, line 54 we use the terminology involvement, as it refers to an active doing on the individual level. As we are not only interest in involvement understood as participation in e.g. patient care, but involvement through the course of disease, we maintain the term involvement.

**Change to Text**:

**Comment 5**:
Conference abstracts and papers and reports regarding policies and strategies in use by professional bodies or organizations will be excluded, please give reason for excluding them.

### Author’s Response: Thank you for your comment. In the methods sections Eligible study designs and studies (P: 4)

we have written that grey literature will be included. Since conference abstracts and papers regarding policies and strategies can be considered as grey literature, we have deleted the sentence

**Change to Text**:

**Comment 6**: Methods - indicate the search terms and the criteria you will use to appraise the selected studies.

Author’s Response: Thank you for your comment. We have created a table 1, that shows the search terms and where the MesH terms are highlighted in bold.

**Change to Text**:

**Comment 7**: Table 1: The mesh search terms are not exhaustive. consider adding registered nurses, registered nurse, practice nurse, license nurses. Also for involvement consider adding participation, communicateon, decision making, care, caring

Author’s Response: Thank you for your suggestions. We have added the suggested terms and have updated the initial search (See table 2)

**Change to Text**: N/A

***Reviewer 3***

**Comment 1**: The scoping review protocol was generally good and presented in line with the PRISMA checklist for scoping review (Tricco et al., 2018). However, the reviewer may consider making changes to the title to include or account for the disease condition highlighted in the body of the scoping review as the participants aspect of the PICO framework. This would enhance title completion and improve readers’ experience.

Author’s Response: Thank you for your comment. We have included the disease conditions as suggested.

# **Change to Text**: Facilitators and barriers towards nurses’ involvement of relatives through the course of disease of individuals with an acquired brain injury or malignant brain tumour.: a scoping review protocol

**Comment 2**: There is still, not a mention of the use of critical appraisal tool for quality appraisal. Also, there is no evidence of the data search flow chart in the protocol. This can improve the pictorial or construct validity of the scoping review.

**Author’s Response**: Thank you for your comment. According to Munn et.al (2018) It is not within the remit of a scoping review to assess	quality of the study or to produce a critically appraised answer to a particular question(1). As Munn states ‘An assessment of methodological limitations or risk of bias of the evidence included within a scoping review is generally not performed’(1). The scoping review methodology is chosen because it is suitable to give an indication of the amount of literature as well as and studies available and further to clarify concepts(1). On page 5 we write that the systematic literature search will be summarized and presented in a PRISMA-ScR flow chart as suggested by JBI. (3)

**Comment 3**: The mention of search databases, the dates of publication of interest, and the key search term provided offers readers insight into how search outcome result where obtained: this is good practice needed as prove of reliability. It also means that research outcome can be reproduced elsewhere

**Author’s Response**: Thank you for your comment. We have inserted a Table 1, that shows search key search terms. In addition, we have provided information on databases and additional sources on page 4, line 136-137 and dates of publication of interest on page 5, line 152-154

**Comment 4**:

Otherwise, the registered protocol is well written in a clear, simple, and concise manner for readers' understanding.

**Author’s Response**: Thank you very much for this comment

1.	Munn Z, Peters MDJ, Stern C, Tufanaru C, McArthur A, Aromataris E. Systematic review or scoping review? Guidance for authors when choosing between a systematic or scoping review approach. BMC Medical Research Methodology. 2018;18(1):143.

2.	Levac D, Colquhoun H, O'Brien KK. Scoping studies: advancing the methodology. Implementation Science. 2010;5(1):69.

3.	Peters MDJ GC, McInerney P, Munn Z, Tricco AC, Khalil, H. . Chapter 11: Scoping Reviews. . In: Aromataris E MZ, editor. JBI Manual for Evidence Synthesis. Adelaide: JBI2020.

4.	Colquhoun HL, Levac D, O'Brien KK, Straus S, Tricco AC, Perrier L, et al. Scoping reviews: time for clarity in definition, methods, and reporting. J Clin Epidemiol. 2014;67(12):1291-4.

***Review report***

1. **Topic:** The topic is clearly writing and significant to undertake the study. It is also scientifically sound as it contains the population to be study and its comparison which is the patients.
2. **Abstract:** The abstract is also well writing and included the registration number of the protocol.
3. **Introduction:** the background is very detailed, informative, and well writing. The rational of the study is also included “to investigate possible facilitators and barriers towards nurses’ involvement of relatives to individuals with acquired brain injury or malignant brain cancer in all settings”. Previous literature used are also referenced.
4. **Methods and materials:** the objective of the study is well writing, specific and in simple terms. The study design aim to be used is systematic review which is recommended to provide highest source of evidence.

- They have stated the inclusion criteria which is “studies looking at nurses caring for patients in acquired brain damage in all settings”. However, the exclusion criteria are not stated in the protocol which might be important to guide those doing the extraction process.
- They have also stated in the methods how search strategy is to be conducted in steps and the sources of information. Data extraction was indicated will be done by two independent extractors and third arbitrator will be involve in case of disagreement.

5. **Data presentation:** it is stated that the data presentation will be done in a tabular form followed by narrative summary.
6. **Overall: I consider the study significant and recommend its commencement because the study is relevant to healthcare professionals to break the barriers of not involving relatives in patients’ management.**
