# Supplementary material for: Facilitators and barriers of relatives’ involvement in nursing care decisions and self-care of patients with acquired brain injury or malignant brain tumour: A scoping review protocol
Source: PLoS One. 2022 Aug 18;17(8):e0273151. doi: 10.1371/journal.pone.0273151 (PMC9387856; doi:10.1371/journal.pone.0273151)
Supplement: Attachment — Submitted filename: PLUS ONE.docx [file pone.0273151.s003.docx]

Based on the initial manuscript provided and the current one submitted, concerns raised previously have been addressed by the authors. However, there is a need for further editing. For example, Line 125: The objective of this scoping review is to identify and map the available evidence concerning on what kind of…Please review the use of prepositions ‘’concerning on what kind of …Authors should review the use of the preposition ''on'' I think it was a typographical error.

An adequate justification was provided for the scoping review, this is critical. Well done!

Given the use of a structured scoping review framework, the different sections of the proposal were guided by the chosen JBI model, which is good. In terms of feasibility, the proposal is feasible as guided by the JBI model, which was emphasized all through the method section. With this, the scoping review will be structured and organized.

I am particularly impressed with the changes in the method section which makes it easier for readers to understand. The expansion of the search outcome and keywords alongside the mention of the different bibliometric databases authors would use inspires trust and transparency in the work.

Overall, I think the authors have done a good job. They should re-read, review, and edit the typographical errors highlighted above. In my opinion, they have done a great job. Well done!
