# Supplementary material for: Facilitators and barriers of relatives’ involvement in nursing care decisions and self-care of patients with acquired brain injury or malignant brain tumour: A scoping review protocol
Source: PLoS One. 2022 Aug 18;17(8):e0273151. doi: 10.1371/journal.pone.0273151 (PMC9387856; doi:10.1371/journal.pone.0273151)
Supplement: Attachment — Submitted filename: 290722 Response to Reviewers.docx [file pone.0273151.s004.docx]

Response to Reviewers

Title: Facilitators and barriers towards nurses’ involvement of relatives in decision making and daily life activities through the course of disease of individuals with an acquired brain injury or malignant brain tumour: A scoping review protocol

New title: Facilitators and barriers of relative’s involvement in nursing care decisions and self-care of patients with malignant brain injury or tumour: a scoping review protocol.

PLOS ONE - PONE-D-21-20624R1.

| **Comments to the Author** |  |
| --- | --- |
| PLOS ONE does not copyedit accepted manuscripts, so the language in submitted articles must be clear, correct, and unambiguous. Any typographical or grammatical errors should be corrected at revision, so please note any specific errors here. | Thank you, the manuscript has been proof-read before re-submission. |
|  |  |
| **Reviewer #2** |  |
| The title is too long: suggesting to use: facilitators and barriers of relatives involvement in nursing care decisions and self-care of patients with brain injury or tumour: a scoping review protocol. | Thank you for the comment. We have changed the title as suggested. |
| It is good that the proposed scoping review will be performed following the Joanna Briggs Institute's (JBI) methodology for scoping reviews but still the authors should have a conceptual or theoretical framework to guide them on possible independent variables (barrier and facilitators) and how how the relate to each other. | Thank you for your nice comment.  We have added the following to the concept section:  The overarching concept of interest for the scoping review is what kind of possible facilitators and barriers towards nurses’ involvement of relatives to individuals with ABI or MBT in all settings. We expect to identify text representation both concepts of facilitators and barriers independently, but also a relationship between the two. |
| Despite the authors point argument, i still belief that the researches need to be appraised before using their results in the study. Poorly perform researches should not inform science. | Thank you for your comment. Scoping reviews are an increasingly popular approach to synthesizing evidence in a number of fields (Munn, 2022). Scoping reviews has the overall aim to examine and map existing literature on a topic to clarify if a systematic review is relevant to conduct. Scoping reviews must be conducted with the same methodological rigor and transparency as e.g., a systematic review (Tricco, 2022) However, conducting an scoping reviews differ from conducting a systematic review because authors do not typically assess the quality of included studies (Levac, 2010). According to Munn (2022) scoping reviews can extend beyond mapping the literature for example identify the types of available evidence in a given field and/or identify key characteristics related to a concept. Since we aim to identify and map all the available evidence concerning possible facilitators and barriers towards nurses’ involvement of relatives through the course of disease of individuals with ABI or MBT in all settings we believe a scoping review is a suitable methodology for this purpose. |
| **Reviewer #3** |  |
| Based on the initial manuscript provided and the current one submitted, concerns raised previously have been addressed by the authors.  However, there is a need for further editing. For example, Line 125: The objective of this scoping review is to identify and map the available evidence concerning on what kind of…Please review the use of prepositions ‘’concerning on what kind of …Authors should review the use of the preposition ''on'' I think it was a typographical error. | Thank you for the acknowledgement.  Thank you for your comment. The aim has been changed: The objective of this scoping review is to identify and map the available evidence concerning possible facilitators and barriers towards nurses’ involvement of relatives through the course of disease of individuals with ABI or MBT in all settings. |
| An adequate justification was provided for the scoping review, this is critical. Well done! | Thank you for your nice comment. |
| Given the use of a structured scoping review framework, the different sections of the proposal were guided by the chosen JBI model, which is good. In terms of feasibility, the proposal is feasible as guided by the JBI model, which was emphasized all through the method section. With this, the scoping review will be structured and organized. | Thank you for your nice comment. |
| I am particularly impressed with the changes in the method section which makes it easier for readers to understand. The expansion of the search outcome and keywords alongside the mention of the different bibliometric databases authors would use inspires trust and transparency in the work. | Thank you for your nice comment. |
| Overall, I think the authors have done a good job. They should re-read, review, and edit the typographical errors highlighted above. In my opinion, they have done a great job. Well done! | Thank you for your nice comment. The manuscript has been proof-read before re-submission. |
